# Metabolic syndrome is independently associated with 1-year readmission in patients with alcohol-associated cirrhosis

**DOI:** 10.3389/fmed.2026.1861502

**Published:** 2026-08-13

**Authors:** Jiarui Zheng, Yuxuan Song, Yanxu Hao, Bo Feng, Yandi Xie

**Affiliations:** 1 Peking University People's Hospital, Peking University Hepatology Institute, Infectious Disease and Hepatology Center of Peking University People's Hospital, Beijing, China; 2 Beijing Key Laboratory of Hepatitis C and Immunotherapy for Liver Diseases, Beijing International Cooperation Base for Science and Technology on NAFLD Diagnosis, Beijing, China; 3 Beijing Jingmei Group General Hospital, Beijing, China

**Keywords:** metabolic syndrome, alcohol-associated cirrhosis, metabolic dysfunction and alcohol-associated liver disease, metabolic dysfunction-associated steatotic liver disease, readmission

## Abstract

**Objectives:**

This study aimed to investigate the relationship between metabolic syndrome (MetS) and the risk of 1-year readmission in patients with alcohol-associated cirrhosis and to identify the independent predictors of this outcome.

**Methods:**

This retrospective cohort study included 379 patients with alcohol-associated cirrhosis who were hospitalized between January 2012 and December 2023. Patients were categorized into two groups according to the presence or absence of MetS. Baseline characteristics, liver function indicators, complications, and causes of readmission were compared. A multivariable logistic regression analysis was used to identify independent predictors of 1-year readmission. Stratified analyses were conducted to evaluate potential effect modification.

**Results:**

Of the 379 patients (98.9% were male patients, mean age 54.1 ± 10.1 years), 40 of them (10.6%) had MetS. Patients with MetS had a significantly higher 1-year readmission rate than those without MetS (65.0% vs. 44.5%, *p* = 0.014). Overall, 177 patients (46.7%) were readmitted within 1 year. The readmitted group had higher rates of MetS (14.7% vs. 6.9%, *p* = 0.014), hepatic encephalopathy (19.2% vs. 10.9%, *p* = 0.023), and variceal bleeding (13.6% vs. 5.0%, *p* = 0.003). Ascites was the most common cause of readmission (55.4%), followed by hepatic encephalopathy (19.8%), hepatocellular carcinoma (14.1%), and variceal bleeding (13.6%). A multivariable analysis confirmed that MetS (OR = 2.38, 95% CI: 1.17–4.83, *p* = 0.017), hepatic encephalopathy, variceal bleeding, and lower alanine aminotransferase (ALT) levels were independent predictors of 1-year readmission. Stratified analyses showed no significant interaction between MetS and the risk of 1-year readmission across different subgroups stratified by age, BMI, hypertension, diabetes, hyperlipidemia, ALT levels, aspartate aminotransferase (ALP) levels, and total bilirubin (TBIL) levels.

**Conclusion:**

Metabolic syndrome is an independent predictor of 1-year readmission in patients with alcohol-associated cirrhosis, influencing the risk of readmission together with factors such as hepatic encephalopathy and variceal bleeding. The interplay between metabolic dysfunction and alcohol-associated cirrhosis warrants further investigation, and our findings underscore the importance of metabolic risk management in this population.

## Introduction

The incidence of alcohol-associated liver disease (ALD) is estimated to affect approximately 5% of the global population (1), and it is the primary cause of liver-related fatalities in numerous countries (2). Due to the extensive at-risk population, alcohol-associated cirrhosis has emerged as the primary reason for liver transplantation among adults in the United States (3, 4) and the primary cause of liver-related mortality on a global scale (5).

Metabolic syndrome (MetS) is a global health issue characterized by a cluster of conditions, including elevated blood pressure, dyslipidemia, hyperglycemia or insulin resistance, and abdominal obesity (6). MetS is frequently observed in individuals with metabolic dysfunction-associated steatotic liver disease (MASLD), formerly known as metabolic-associated fatty liver disease (MAFLD). The 2023 multi-society Delphi consensus recommended replacing “fatty” with “steatotic” to reduce stigma and improve diagnostic precision (7). MetS has been identified as an independent risk factor for liver-related complications, fibrosis progression, and hepatocellular carcinoma (HCC) (8, 9).

Alcohol consumption and metabolic dysfunction frequently co-occur and exert synergistic effects on liver injury (10). The 2023 AASLD guidelines introduced the term MetALD to describe patients with both significant alcohol consumption and metabolic risk factors (7), recognizing the dynamic interplay between these two etiologies. The presence of MetS can accelerate cirrhosis progression and increase the risk of hepatic decompensation, potentially leading to higher hospital readmission rates (10–12). However, the specific impact of MetS on readmission risk in patients with alcohol-associated cirrhosis remains insufficiently explored.

Therefore, this study aimed to investigate whether MetS is an independent predictor of 1-year hospital readmission among individuals with alcohol-associated cirrhosis and to explore the underlying mechanisms, thereby offering novel perspectives and approaches for clinical management.

## Methods

### Study population

**Figure 1:**
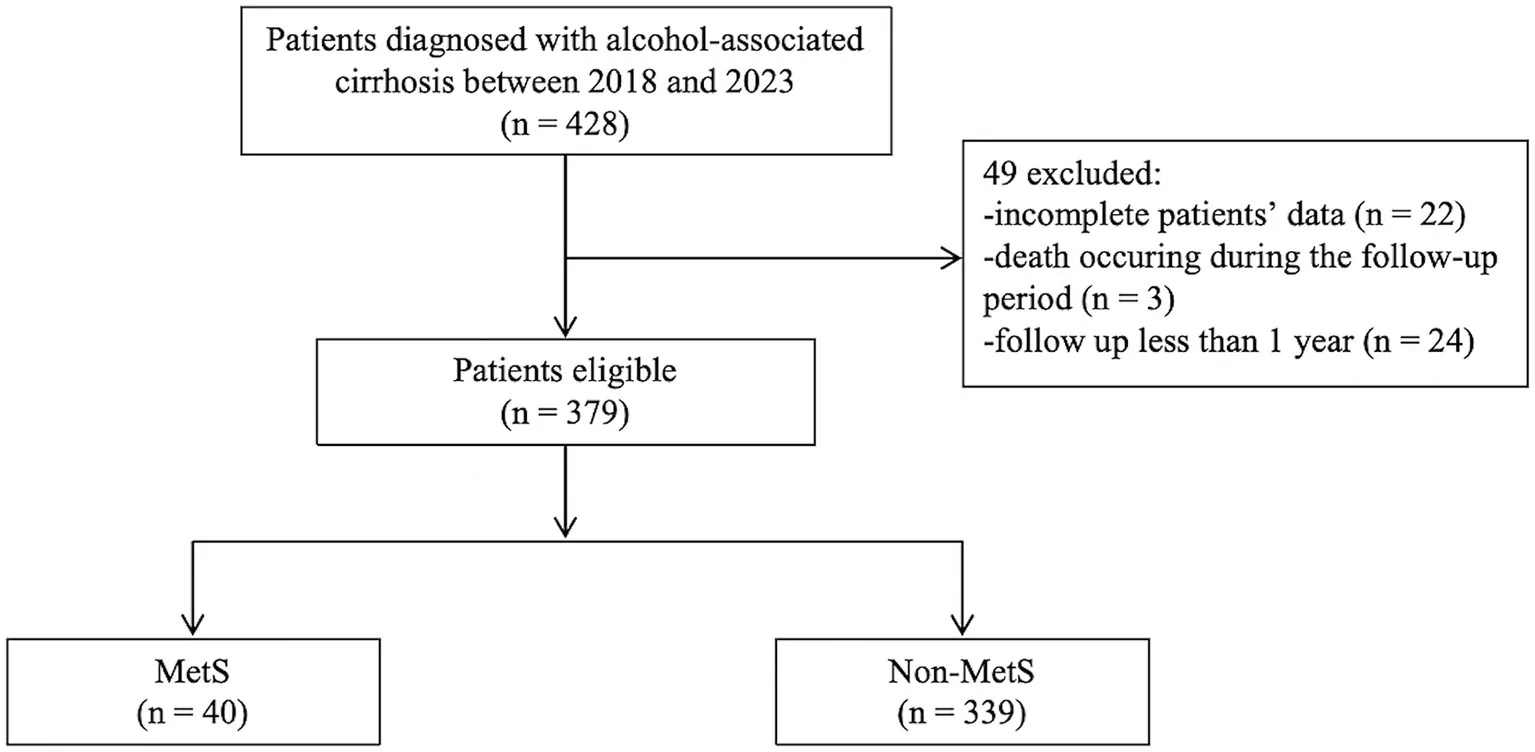
Flowchart of selecting patients with alcohol-associated cirrhosis. MetS, Metabolic syndrome.

Patients hospitalized between January 2012 and December 2023 with a diagnosis of alcohol-associated cirrhosis were retrospectively enrolled. The inclusion criteria were as follows: (a) age ≥ 18 years; (b) availability of complete data on all study variables, defined as the presence of required demographic (age, sex, body mass index [BMI]), laboratory (alanine aminotransferase [ALT], alkaline phosphatase [ALP], aspartate aminotransferase [AST], total bilirubin [TBIL], creatinine, lipids, and glucose) and clinical outcome (ascites, hepatic encephalopathy, variceal bleeding, HCC, and 1-year readmission) data without missing values for the primary analysis variables; (c) admission for a liver-related condition with an established diagnosis of alcohol-associated cirrhosis; and (d) a follow-up period of at least 1 year. The exclusion criteria were as follows: (a) incomplete data on key variables (including missing data on MetS components, admission diagnosis, or readmission status) and (b) death occurring during the follow-up period (Figure 1).

The diagnosis of alcohol-associated cirrhosis was established based on the following criteria: (1) documented history of significant alcohol consumption (>40 g/day in men, >20 g/day in women for ≥5 years); (2) clinical, laboratory, and imaging evidence of cirrhosis (e.g., liver stiffness measurement, ultrasonography, computed tomography, or transient elastography findings consistent with cirrhosis); and (3) histopathological confirmation from liver biopsy, when available. Diagnoses were confirmed by an independent review of medical records, including clinical notes, laboratory data, and imaging reports, by two hepatologists.

The Institutional Ethics Committee of Peking University People’s Hospital approved the study protocol (No. 2024PHB047-001). All procedures were conducted in accordance with the ethical standards established in the 1975 Declaration of Helsinki and its subsequent 1983 amendment. Given the retrospective nature of this study, the requirement for individual informed consent was formally waived.

### Clinical characteristics

We retrieved information on patient hospitalizations from our hospital’s electronic medical records. Patients’ characteristics included age, gender, BMI, laboratory parameters (including platelet count, ALT, ALP, AST, TBIL, albumin, and triglycerides), co-morbidities (hypertension, diabetes, hyperlipidemia, and chronic kidney disease), and complications (ascites, hepatic encephalopathy, variceal bleeding, and HCC). ALT levels were considered elevated if they exceeded 30 U/L in men and 19 U/L in women. Elevated AST levels were defined as those exceeding 40 U/L, while ALP levels were considered elevated if they surpassed 120 U/L. Additionally, TBIL levels were deemed elevated if they were greater than 17.1 μmol/L.

### Metabolic syndrome definition

We assessed BMI, blood pressure, glycosylated hemoglobin (HbA1c), fasting plasma glucose (FPG), total cholesterol, plasma triglycerides, and high- and low-density lipoproteins (HDL and LDL). For patients admitted with ascites, BMI was calculated using body weight measured at discharge (within 24 h before discharge) rather than at admission, because the recorded body weight primarily reflected the patients’ dry weight and provided a more accurate estimation of their true BMI. Dyslipidemia was defined as HDL cholesterol <1.30 mmol/L for women and <1.00 mmol/L for men and/or triglycerides ≥1.7 mmol/L. Hypertension was defined as systolic blood pressure >130 mmHg, diastolic blood pressure >85 mmHg, or receiving treatment for previously diagnosed hypertension. Elevated FPG was defined as FPG > 100 mg/dL (5.6 mmol/L) or previously diagnosed type 2 diabetes. Central obesity was defined as waist circumference ≥90 cm for men and ≥80 cm for women, and if BMI exceeded 30 kg/m^2^, abdominal obesity was presumed, eliminating the need for waist measurement. MetS was defined according to the International Diabetes Federation (IDF) criteria in this study as central obesity along with any two of the following: dyslipidemia, hypertension, and elevated FPG or previously diagnosed type 2 diabetes (13, 14).

### Study outcomes

The primary endpoint of our study was defined as the first liver-related readmission within 1 year after the patients’ initial hospital admission. Liver-related readmission was considered to occur due to any of the following conditions: ascites, hepatic encephalopathy, variceal bleeding, or HCC.

### Statistical analysis

The normality of the data was assessed using the Kolmogorov–Smirnov test. Patients’ baseline characteristics were summarized as follows: Normally distributed continuous data were presented as mean ± standard deviation (SD), while non-normally distributed continuous data were described using the median [interquartile range (IQR)]. Categorical variables were reported as counts (%). To determine significant differences between groups, the Mann–Whitney U test or the independent samples *t*-test was used for continuous variables, and the chi-squared test was used for categorical variables. Univariate and multivariate analyses were performed to identify predictors of 1-year readmission among patients with alcohol-associated cirrhosis. Subgroup analyses were conducted to investigate the association between MetS and 1-year readmission according to age, BMI, hypertension, diabetes, ALT level (<ULN, ≥ULN), ALP level (<ULN, ≥ULN), and TBIL level (<ULN, ≥ULN). Given that ascites can artificially increase body weight and thus influence BMI-based assessment of central obesity, we performed a stratified analysis of the association between MetS and readmission risk by baseline ascites status. Statistical significance was determined at a *p*-value of less than 0.05. Statistical analysis was performed using the R software package (version 4.1.1, R Foundation for Statistical Computing, Vienna, Austria).

## Results

### Baseline characteristics by MetS status

In total, 379 patients with alcohol-associated cirrhosis were enrolled in this study, with the majority being male (98.9%) and having a mean age of 54.1 ± 10.1 years. Liver-related causes for first admission were ascites (60.2%), hepatic encephalopathy (14.8%), HCC (12.1%), and variceal bleeding (9.0%), while 29.8% of patients had none of these complications. Among the enrolled individuals, 40 (10.6%) had MetS, among whom the proportions of overweight, obesity, hypertension, diabetes, and hyperlipidemia were 62.5, 32.5, 87.5, 82.5, and 25.0%, respectively. In comparison with individuals without MetS, those with MetS exhibited a lower prevalence of ascites (16 [40.0%] vs. 212 [62.5%], *p* = 0.006), lower ALP levels [99.0 (65.8, 140.8) U/L vs. 121.0 (90.0, 175.0) U/L, *p* = 0.004], lower TBIL levels [25.5 (15.4, 51.4) μmol/L vs. 35.7 (21.3, 70.8) μmol/L, *p* = 0.035], and *p = 0.037*, as well as an increased risk of the 1-year readmission (26 [65.0%] vs. 151 [44.5%], *p =* 0.014) (Table 1).

**Table 1:** Baseline characteristics of patients by MetS.

Variables	All patients (*n* = 379)	MetS (*n* = 40)	Non-MetS (*n* = 339)	*p*-value
Age (years)	54.1 ± 10.1	56.5 ± 9.3	53.9 ± 10.2	0.123
<60	279 (73.6)	26 (65.0)	253 (74.6)	0.191
≥60	100 (26.4)	14 (35.0)	86 (25.4)	
Male (n,%)	375 (98.9)	39 (97.5)	336 (99.1)	0.361
BMI (kg/m^2^)	24.0 ± 3.8	27.0 ± 2.8	23.7 ± 3.8	< 0.001
<24	182 (48.0)	2 (5.0)	180 (53.1)	< 0.001
24–28	145 (38.3)	25 (62.5)	120 (35.4)	
≥28	52 (13.7)	13 (32.5)	39 (11.5)	
Alcohol consumption (years)	26.3 ± 11.1	29.0 ± 9.4	26.0 ± 11.3	0.096
Alcohol intake (g/day)	139.5 ± 90.6	112.1 ± 86.3	142.8 ± 90.7	0.043
Co-morbidities (n, %)				
Hypertension	121 (31.9)	35 (87.5)	86 (25.4)	< 0.001
Diabetes	109 (28.8)	33 (82.5)	76 (22.4)	< 0.001
Hyperlipidemia	26 (6.9)	10 (25.0)	16 (4.7)	< 0.001
Chronic kidney disease	38 (10.0)	5 (12.5)	33 (9.7)	0.577
Complications (n, %)				
Ascites	228 (60.2)	16 (40.0)	212 (62.5)	0.006
Hepatic encephalopathy	56 (14.8)	3 (7.5)	53 (15.6)	0.170
Variceal bleeding	34 (9.0)	3 (7.5)	31 (9.1)	0.571
Hepatocellular carcinoma	46 (12.1)	6 (15.0)	40 (11.8)	0.607
Laboratory parameters				
Platelet count (× 10^9^/L)	83.0 (54.0, 130.5)	105.5 (69.0, 141.2)	81.0 (53.5, 127.5)	0.074
ALT (U/L)	30.0 (20.0, 51.5)	29.0 (19.0, 55.2)	30.0 (21.0, 51.0)	0.588
<ULN	178 (47.0)	20 (50.0)	158 (46.6)	0.684
≥ULN	201 (53.0)	20 (50.0)	181 (53.4)	
AST (U/L)	55.0 (36.0, 83.0)	52.0 (30.2, 68.5)	55.0 (37.0, 86.5)	0.063
<ULN	117 (30.9)	18 (45.0)	99 (29.2)	0.041
≥ULN	262 (69.1)	22 (55.0)	240 (70.8)	
ALP (U/L)	120.0 (87.5, 172.5)	99.0 (65.8, 140.8)	121.0 (90.0, 175.0)	0.004
<ULN	205 (54.1)	26 (65.0)	179 (52.8)	0.143
≥ULN	174 (45.9)	14 (35)	160 (47.2)	
TBIL (μmol/L)	34.3 (20.4, 68.6)	25.5 (15.4, 51.4)	35.7 (21.3, 70.8)	0.035
<ULN	72 (19.0)	11 (27.5)	61 (18)	0.147
≥ULN	307 (81.0)	29 (72.5)	278 (82)	
Albumin (g/L)	32.9 ± 17.1	32.9 ± 6.6	32.9 ± 17.9	0.987
Triglyceride (mg/dL)	1.2 ± 0.8	2.0 ± 1.6	1.1 ± 0.6	< 0.001
MELD score	9.9 ± 6.2	8.0 ± 6.4	10.2 ± 6.2	0.037
1-year readmission (*n*,%)	177 (46.7)	26 (65.0)	151 (44.5)	0.014

Categorical values are presented as n (%). Continuous variables are presented as the mean ± SEs. Elevated alanine aminotransferase (ALT) level was defined as ALT > 30 U/L in men or > 19 U/L in women. Elevated aspartate aminotransferase (AST) level was defined as AST > 40 U/L. Elevated alkaline phosphatase (ALP) level was defined as ALP > 120 U/L. Elevated total bilirubin (TBIL) level was defined as TBIL > 17.1 μmol/L. MetS, metabolic syndrome; BMI, Body mass index; ALT, Alanine aminotransferase; AST, Aspartate aminotransferase; ALP, Alkaline phosphatase; TBIL, Total bilirubin; MELD, Model for end-stage liver disease.

### Baseline characteristics by 1-year readmission

Of the overall cohort, 177 patients (46.7%) were readmitted within 1 year. The readmitted group had a higher mean BMI (24.4 ± 4.3 vs. 23.7 ± 3.4 kg/m^2^, *p* = 0.047), a higher platelet count [89.0 (61.0, 137.0) vs. 74.5 (50.2, 116.8) × 10^9^/L, *p* = 0.007], and lower ALT [28.0 (19.0, 45.0) vs. 36.0 (22.0, 65.0) U/L, *p* = 0.014] and ALP [113.0 (85.0, 164.0) vs. 126.0 (92.0, 178.8) U/L, *p* = 0.029] levels. Regarding comorbidities and complications, readmitted patients had significantly higher rates of MetS (14.7% vs. 6.9%, *p* = 0.014), hepatic encephalopathy (19.2% vs. 10.9%, *p* = 0.023), and variceal bleeding (13.6% vs. 5.0%, *p* = 0.003) (Table 2).

**Table 2:** Baseline characteristics of patients by 1-year readmission.

Variables	All patients (*n* = 379)	Readmission (*n* = 177)	Non-readmission (*n* = 202)	*P*-value
Age (years)	54.1 ± 10.1	54.3 ± 9.2	54.0 ± 10.9	0.817
<60	279 (73.6)	130 (73.4)	149 (73.8)	0.944
≥60	100 (26.4)	47 (26.6)	53 (26.2)	
Male (n,%)	375 (98.9)	174 (98.3)	201 (99.5)	0.343
BMI (kg/m^2^)	24.0 ± 3.8	24.4 ± 4.3	23.7 ± 3.4	0.047
<24	182 (48.0)	83 (46.9)	99 (49.0)	0.123
24–28	145 (38.3)	63 (35.6)	82 (40.6)	
≥28	52 (13.7)	31 (17.5)	21 (10.4)	
Alcohol consumption (years)	26.3 ± 11.1	27.6 ± 11.2	25.2 ± 10.9	0.037
Alcohol intake (g/day)	139.5 ± 90.6	134.4 ± 91.2	144.0 ± 90.1	0.301
(*n*, %)				
Hypertension	121 (31.9)	69 (39)	52 (25.7)	0.006
Diabetes	109 (28.8)	56 (31.6)	53 (26.2)	0.246
Hyperlipidemia	26 (6.9)	13 (7.3)	13 (6.4)	0.727
MetS	40 (10.6)	26 (14.7)	14 (6.9)	0.014
Chronic kidney disease	38 (10.0)	13 (7.3)	25 (12.4)	0.104
Complications (n, %)				
Ascites	228 (60.2)	105 (59.3)	123 (60.9)	0.756
Hepatic encephalopathy	56 (14.8)	34 (19.2)	22 (10.9)	0.023
Variceal bleeding	34 (9.0)	24 (13.6)	10 (5.0)	0.003
Hepatocellular carcinoma	46 (12.1)	24 (13.6)	22 (10.9)	0.427
Laboratory parameters				
Platelet count (× 10^9^/L)	83.0 (54.0, 130.5)	89.0 (61.0, 137.0)	74.5 (50.2, 116.8)	0.007
ALT (U/L)	30.0 (20.0, 51.5)	28.0 (19.0, 45.0)	36.0 (22.0, 65.0)	0.014
<ULN	178 (47.0)	95 (53.7)	83 (41.1)	
≥ULN	201 (53.0)	82 (46.3)	119 (58.9)	
AST (U/L)	55.0 (36.0, 83.0)	54.0 (34.0, 79.0)	58.5 (39.0, 88.5)	0.101
<ULN	117 (30.9)	62 (35.0)	55 (27.2)	
≥ULN	262 (69.1)	115 (65.0)	147 (72.8)	
ALP (U/L)	120.0 (87.5, 172.5)	113.0 (85.0, 164.0)	126.0 (92.0, 178.8)	0.029
<ULN	205 (54.1)	105 (59.3)	100 (49.5)	0.056
≥ULN	174 (45.9)	72 (40.7)	102 (50.5)	
TBIL (μmol/L)	34.3 (20.4, 68.6)	30.2 (17.5, 66.3)	35.8 (22.7, 69.0)	0.062
<ULN	72 (19.0)	41 (23.2)	31 (15.3)	0.053
≥ULN	307 (81.0)	136 (76.8)	171 (84.7)	
Albumin (g/L)	32.9 ± 17.1	32.0 ± 5.9	33.6 ± 22.7	0.363
Triglyceride (mg/dL)	1.2 ± 0.8	1.2 ± 0.9	1.1 ± 0.7	0.26
MELD score	9.9 ± 6.2	9.4 ± 6.2	10.4 ± 6.3	0.102

Categorical values are presented as n (%). Continuous variables are presented as the mean ± SEs. Elevated alanine aminotransferase (ALT) level was defined as ALT > 30 U/L in men or > 19 U/L in women. Elevated aspartate aminotransferase (AST) level was defined as AST > 40 U/L. Elevated alkaline phosphatase (ALP) level was defined as ALP > 120 U/L. Elevated total bilirubin (TBIL) level was defined as TBIL > 17.1 μmol/L. MetS: etabolic syndrome; BMI: Body mass index; ALT: Alanine aminotransferase; AST: Aspartate aminotransferase; ALP: Alkaline phosphatase; TBIL: Total bilirubin; MELD: Model for end-stage liver disease.

### Causes of readmission

**Figure 2:**
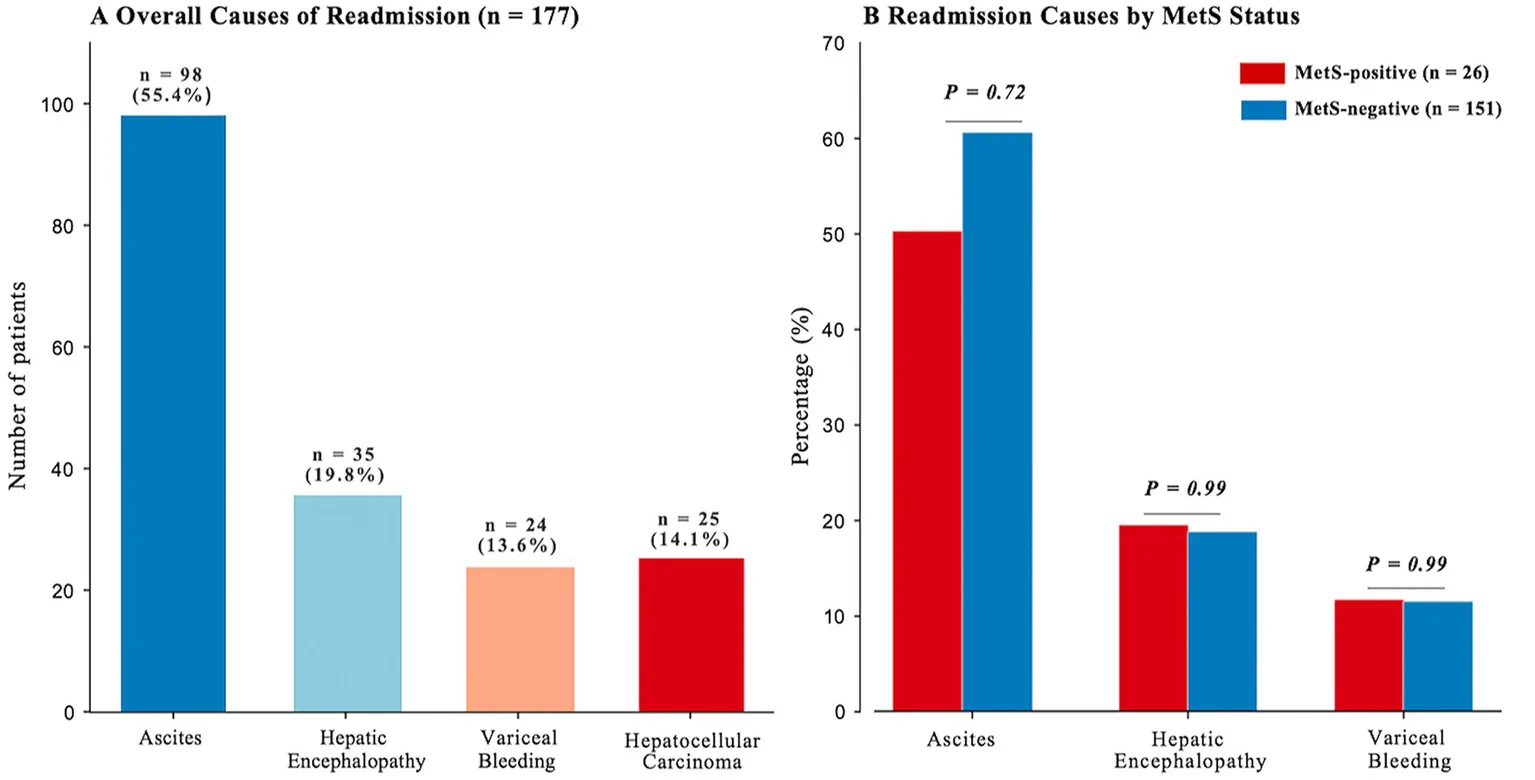
Overall causes of 1-year readmission and stratified by MetS status. **(A)** Overall causes of readmission. **(B)** Readmission causes by MetS status. MetS, Metabolic syndrome.

Among the 177 readmitted patients, the most common cause of readmission was ascites (98 patients, 55.4%), followed by hepatic encephalopathy (35 patients, 19.8%), HCC (25 patients, 14.1%), and variceal bleeding (24 patients, 13.6%) (Figure 2A). A total of 30 patients (16.6%) experienced a new decompensation event that was not present at baseline. When stratified by MetS status, the distribution of readmission causes was similar between MetS-positive and MetS-negative patients: ascites (50.2% vs. 60.4%, *p* = 0.72), hepatic encephalopathy (20.1% vs. 19.6%, *p* = 0.99), and variceal bleeding (14.0% vs. 13.3%, *p* = 0.99), suggesting that MetS increases the overall risk of liver-related readmission without preferentially affecting specific decompensation types (Figure 2B).

### Predictors of 1-year readmission

In the univariate analysis, BMI, hypertension, MetS, ascites, hepatic encephalopathy, variceal bleeding, and ALT levels were significantly associated with 1-year readmission (Table 3). Considering that the diagnosis of MetS includes BMI and hypertension, we only included MetS in the multivariate analysis, which showed that MetS (OR = 2.38, 95% CI: 1.17–4.83, *p* = 0.017), hepatic encephalopathy (OR = 2.19, 95% CI: 1.19–4.05, *p* = 0.012), variceal bleeding (OR = 2.79, 95% CI: 1.26–6.15, *p* = 0.011), and ALT levels (OR = 0.61, 95% CI: 0.40–0.94, *p* = 0.026) were independently associated with 1-year readmission. In the sensitivity analysis, we additionally adjusted for age, gender, MetS, hepatic encephalopathy, variceal bleeding, and ALT levels and established three additional models: Model 1 included hypertension on the basis of the aforementioned variables, Model 2 included BMI, and Model 3 included both BMI and hypertension. The multivariate analysis showed that, in Model 2, after adjusting for BMI, there was a statistical correlation between MetS and 1-year readmission (OR = 2.3, 95% CI: 1.13–4.70, *p* = 0.022). However, after adjusting for hypertension in Models 2 and 3, regardless of whether BMI was included, there was no significant relationship between MetS and 1-year readmission (Table 4).

**Table 3:** Predictors of 1-year readmission in univariable and multivariable logistic regression analyses.

Variables	Univariable		Multivariable	
	OR (95%CI)	*P*-value	OR (95%CI)	*P*-value
Age (years)				
<60	1		1	
≥60	1.02 (0.64–1.61)	0.944	0.89 (0.55–1.45)	0.643
Male (n,%)	0.29 (0.03–2.8)	0.284	0.32 (0.03–3.23)	0.332
BMI (kg/m2)				
<24	1			
≥28	1.83 (1.01–3.32)	0.047		
Hypertension				
No	1			
Yes	1.84 (1.19–2.85)	0.006		
Diabetes				
No	1			
Yes	1.3 (0.83–2.03)	0.247		
Hyperlipidemia				
No	1			
Yes	1.15 (0.52–2.56)	0.727		
MetS				
No	1		1	
Yes	2.31 (1.17–4.58)	0.016	2.38 (1.17–4.83)	0.017
Chronic kidney disease				
No	1			
Yes	0.56 (0.28–1.13)	0.107		
Ascites				
No	1			
Yes	0.94 (0.62–1.41)	0.756		
Hepatic encephalopathy				
No	1		1	
Yes	1.95 (1.09–3.47)	0.024	2.19 (1.19–4.05)	0.012
Variceal bleeding				
No	1		1	
Yes	3.01 (1.4–6.49)	0.005	2.79 (1.26–6.15)	0.011
ALT (IU/L)				
<ULN	1		1	
≥ULN	0.6 (0.4–0.9)	0.015	0.61 (0.4–0.94)	0.026
AST (IU/L)				
<ULN	1			
≥ULN	0.69 (0.45–1.07)	0.102		
ALP (IU/L)				
<ULN	1			
≥ULN	0.67 (0.45–1.01)	0.056		
TBIL (μmol/L)				
<ULN	1			
≥ULN	0.6 (0.36–1.01)	0.054		
Albumin (g/L)	0.99 (0.97–1.01)	0.435		
MELD score	0.97 (0.94–1.01)	0.103		

Multivariable analysis was adjusted for age, gender, hepatic encephalopathy, variceal bleeding, ALT levels, and MetS. Elevated alanine aminotransferase (ALT) level was defined as ALT> 30 U/L in men or > 19 U/L in women. Elevated aspartate aminotransferase (AST) level was defined as AST > 40 U/L. Elevated alkaline phosphatase (ALP) level was defined as ALP > 120 U/L. Elevated total bilirubin (TBIL) level was defined as TBIL > 17.1 μmol/L. MetS: Metabolic syndrome; BMI, Body mass index; ALT, Alanine aminotransferase; AST, Aspartate aminotransferase; ALP, Alkaline phosphatase; TBIL, Total bilirubin; MELD, Model for end-stage liver disease.

**Table 4:** Sensitivity analysis of predictors of 1-year readmission in multivariable logistic regression analysis in three models.

Variables	Model 1		Model 2		Model 3	
	OR (95% CI)	*P*-value	OR (95% CI)	*P*-value	OR (95% CI)	*P*-value
Age (years)						
<60	1		1		1	
≥60	0.88 (0.55–1.43)	0.61	0.94 (0.58–1.52)	0.81	0.9 (0.56–1.46)	0.677
Male (*n*, %)	0.27 (0.03–2.77)	0.269	0.31 (0.03–3.15)	0.32	0.28 (0.03–2.91)	0.287
BMI (kg/m2)						
<24	—	—	1		1	
≥28	—	—	1.57 (0.84–2.94)	0.158	1.45 (0.77–2.73)	0.251
Hypertension						
No	1		—	—	1	
Yes	1.59 (0.97–2.61)	0.063	—	—	1.53 (0.93–2.51)	0.096
MetS						
No	1		1		1	
Yes	1.9 (0.89–4.08)	0.099	2.3 (1.13–4.7)	0.022	1.82 (0.84–3.93)	0.128
Hepatic encephalopathy						
No	1		1		1	
Yes	2.02 (1.1–3.7)	0.024	2 (1.09–3.67)	0.025	2.03 (1.1–3.72)	0.023
Variceal bleeding						
No	1		1		1	
Yes	2.88 (1.31–6.34)	0.009	2.96 (1.35–6.5)	0.007	2.89 (1.31–6.37)	0.008
ALT (IU/L)						
<ULN	1		1		1	
≥ULN	0.58 (0.38–0.88)	0.011	0.59 (0.39–0.9)	0.015	0.59 (0.39–0.91)	0.016

Beyond age, gender, hepatic encephalopathy, variceal bleeding, ALT levels, and MetS, Model 1 adjusted for hypertension, Model 2 adjusted for BMI, and Model 3 adjusted for hypertension and BMI. Elevated alanine aminotransferase (ALT) level was defined as ALT > 30 U/L in men or > 19 U/L in women. Elevated aspartate aminotransferase (AST) level was defined as AST > 40 U/L. Elevated alkaline phosphatase (ALP) level was defined as ALP > 120 U/L. Elevated total bilirubin (TBIL) level was defined as TBIL > 17.1 μmol/L. MetS, Metabolic syndrome; BMI, Body mass index; ALT, Alanine aminotransferase; AST, Aspartate aminotransferase; ALP, Alkaline phosphatase; TBIL, Total bilirubin; MELD, Model for end-stage liver disease.

### Subgroup analysis of factors influencing the association between MetS and the 1-year readmission

**Figure 3:**
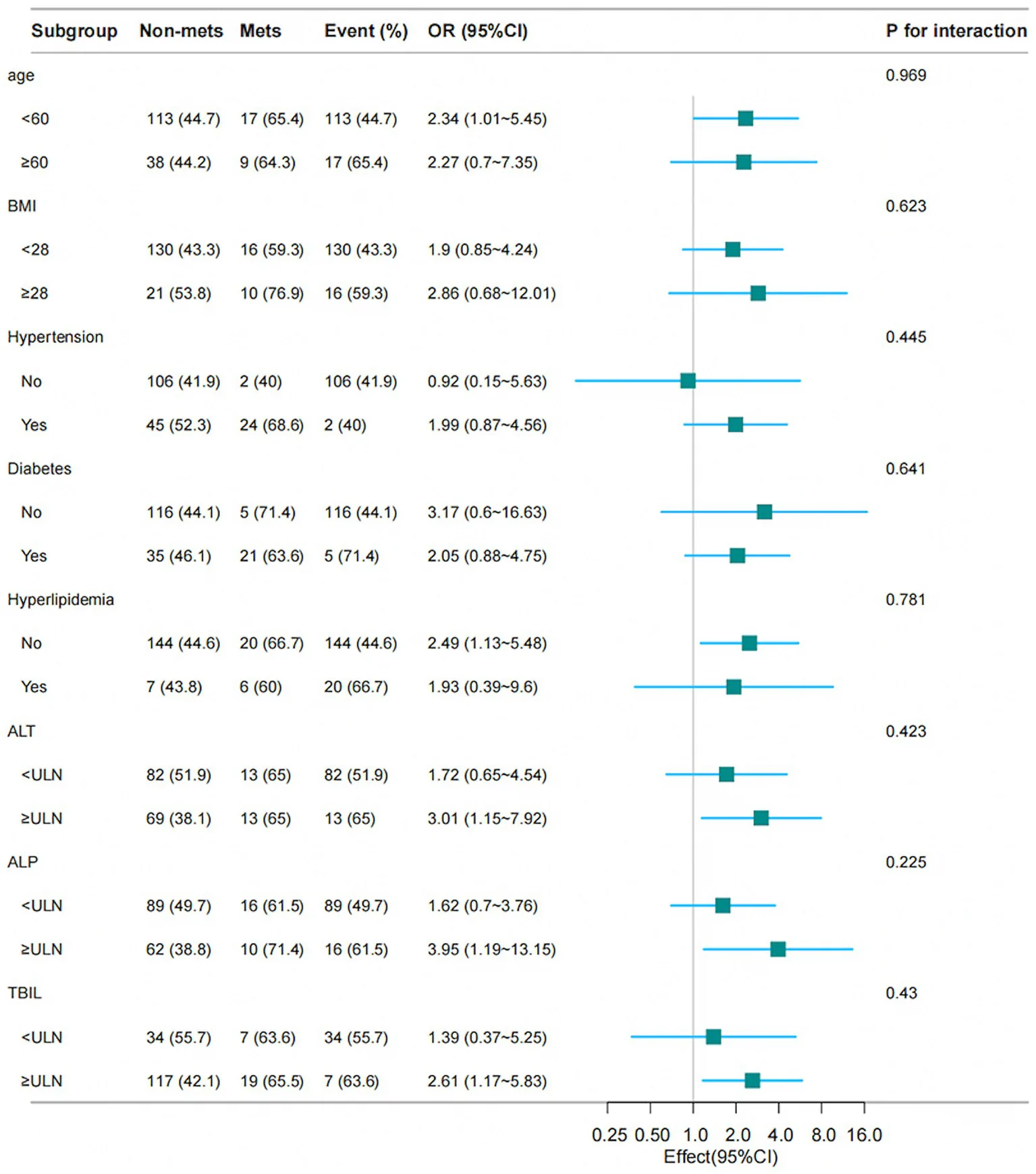
Effect size of MetS on 1-year readmission in the age, BMI, hypertension, diabetes, hyperlipidemia, ALT, ALP, and TBIL subgroups. OR, odds ratio; CI, confidence interval.

To determine potential modifications in the relationship between MetS and 1-year readmission, stratified analyses were conducted across various subgroups. No significant interactions were observed among various patient subgroups after stratifying by age, BMI, hypertension, diabetes, hyperlipidemia, ALT level, ALP level, and TBIL level (all *p* for interaction > 0.05), suggesting that the effect of MetS on readmission risk was consistent across these patient subgroups (Figure 3).

Given that ascites can artificially increase body weight and thereby influence the BMI-based surrogate for central obesity (BMI ≥ 30 kg/m^2^ in lieu of waist circumference ≥90 cm for men, ≥80 cm for women according to IDF criteria), we performed a stratified analysis of the association between MetS and readmission risk by baseline ascites status. In patients without ascites, MetS was significantly associated with readmission (OR = 3.18, 95% CI: 1.23–8.20, *p* = 0.024). In those with ascites, the association showed a similar direction but did not reach statistical significance (OR = 1.55, 95% CI: 0.56–4.33, *p* = 0.556), with no significant interaction between MetS and ascites (*p* for interaction = 0.315), indicating that the MetS–readmission relationship was not modified by ascites status.

## Discussion

In this large retrospective cohort study of 379 patients with alcohol-associated cirrhosis, we found that MetS was independently associated with an increased risk of 1-year readmission. This association remained robust after adjusting for ascites, individual MetS components, and other potential confounding variables. Our findings highlight the prognostic significance of MetS in alcohol-associated cirrhosis, a population in which metabolic risk factors have historically received less clinical attention than those in MASLD.

The dynamic trajectory of steatotic liver diseases—encompassing MASLD, MetALD, and ALD—has been increasingly recognized (15). Patients with both significant alcohol consumption and metabolic risk factors are currently classified under the term MetALD according to the 2023 multi-society consensus (7). In our cohort, of the 40 patients (10.6%) with MetS, 12 (30%) had alcohol consumption within the MetALD range (210–420 g/week [30–60 g/day] for men and 140–350 g/week [20–50 g/day] for women), while the remaining 28 (70%) exceeded the upper threshold and were best characterized as ALD with concurrent metabolic comorbidity. Nevertheless, all MetS-positive patients exhibited a dual-etiology pathophysiology—metabolic dysfunction combined with heavy alcohol intake—that aligns with the clinical concept of the MetALD spectrum. This dual-etiology subgroup warrants particular attention, as the interplay between alcohol and metabolic overload may accelerate disease progression beyond what either factor alone would produce (16, 17). The dynamic nature of these conditions implies that patients may transition between diagnostic categories over time depending on changes in alcohol consumption and metabolic status, underscoring the need for longitudinal monitoring and integrated management strategies.

According to our analysis, patients with MetS exhibited a heightened likelihood of being readmitted within 1 year compared with those without MetS (65.0% vs. 44.5%, *p* = 0.014), which is consistent with prior research indicating a robust association between MetS and adverse outcomes in patients with chronic liver diseases (18, 19). The elevated readmission rate among individuals with MetS may be attributed to several contributing factors. These factors include the exacerbation of liver damage due to key components of MetS, such as obesity, insulin resistance, and hypertension. Moreover, the frequent coexistence of MetS and high-risk alcohol consumption suggests that the pathogenic mechanisms underlying both MASLD and ALD often act synergistically to promote disease progression. MASLD and ALD share similar histological characteristics and numerous overlapping pathogenic pathways (12, 20), as well as a common genetic background (e.g., GPAM, MBOAT7, HSD17B13, PNPLA3, TM6SF2, and APOE) (21, 22). The precise mechanisms driving the interaction between alcohol consumption and metabolic dysfunction remain not fully elucidated. Nevertheless, these interactions could be related to the cumulative impact on oxidative stress (23, 24), mitochondrial dysfunction, CYP2E1 activity (25, 26), hepatic stellate cell activation (27), alterations in gut microbiota along with enhanced intestinal permeability (27–29), abnormal lipid metabolism, and adipocyte dysfunction, which leads to increased lipolysis and the release of proinflammatory factors (27, 30, 31).

Patients with MetS in our study exhibited lower levels of AST, ALP, TBIL, and a lower MELD score than those without MetS. These findings may suggest that MetS may exert a liver-protective effect, which is counterintuitive given the known detrimental effects of MetS on liver health. In fact, individuals with MetS often receive more aggressive management of their metabolic conditions, regardless of lifestyle modifications or pharmacologic interventions such as the use of statins, aspirin, and metformin (32–36), which could mitigate liver damage to some extent. Further large-scale cohort studies are needed to confirm this finding.

The predominant cause of readmission in our cohort was ascites (55.4%), consistent with its role as the most common decompensating event in cirrhosis. Importantly, MetS did not appear to preferentially increase the risk of any specific type of decompensation, suggesting a general effect on cirrhosis progression rather than a pathway-specific mechanism. In the sensitivity analysis, after adjusting for hypertension with or without adjusting for BMI, there was no significant association between MetS and 1-year readmission, suggesting that hypertension may play a key role in the association between MetS and readmission, which can increase the probability of variceal bleeding due to rupture of esophageal and gastric varices. Furthermore, in addition to MetS, our study identified hepatic encephalopathy and variceal bleeding as independent predictors of 1-year readmission. These results are consistent with previous research, which has long recognized these complications as significant contributors to hospital readmission and mortality among patients with cirrhosis (37–39).

The independent association between lower ALT levels and increased 1-year readmission is also noteworthy. ALT is frequently used as an indicator to assess the activity and severity of liver injury in clinical practice. In ALD, AST levels are typically higher than ALT levels, the AST/ALT ratio is often greater than 2, and this ratio has been associated with the degree of hepatocyte damage (5). Some studies have found that lower ALT levels within the normal range are associated with a higher risk of all-cause mortality (40, 41), and some underlying mechanisms may potentially explain the association between the lower ALT level and adverse endpoints: First, ALT functions as an enzyme that is crucial for converting L-alanine and *α*-ketoglutarate into pyruvate and glutamic acid in organs such as the liver, kidneys, skeletal muscle, brain, and heart. Low ALT levels may reflect diminished catalytic capacity, which could affect essential metabolic processes, including gluconeogenesis and amino acid metabolism (42, 43). Second, low ALT levels may reflect a deficiency in vitamin B6. Studies have shown that a deficiency in vitamin B6 may increase the risk of neurocognitive impairment, depression, immune dysfunction, and cardiovascular disease (44, 45).

Several biomarkers and non-invasive tests have emerged for risk stratification in ALD. The enhanced liver fibrosis (ELF) test has been validated for predicting liver-related outcomes in patients with chronic liver disease, including ALD (46). Additionally, predictive biomarkers, such as platelet count, MELD score, and albumin levels for decompensation in individuals with compensated cirrhosis, have been investigated and may improve risk stratification (47). However, our study primarily focused on patients with alcohol-associated cirrhosis, many of whom may have already developed decompensated cirrhosis, and our results showed that the MELD score and albumin were not associated with 1-year readmission. Future studies incorporating these biomarkers alongside MetS assessment could refine the prediction of readmission risk in patients with alcohol-associated cirrhosis and facilitate personalized monitoring strategies.

This study has several limitations. First, the limited sample size constrained our capacity to thoroughly examine the long-term readmission rates associated with MetS in patients with alcohol-associated cirrhosis. Second, the retrospective design may have resulted in selection bias and restricted the causal inferences that can be drawn from the data. Third, the vast majority of patients included in this study were male, which introduces a sex bias and may limit the generalizability of the study results. However, this finding is consistent with the actual situation in China, where the proportion of male patients with alcohol-associated cirrhosis was much higher than that of female patients. Fourth, because of the limited number of deaths in our cohort, we could not examine the association between MetS and mortality among individuals with alcohol-associated cirrhosis. Fifth, ELF test scores and other novel biomarkers were not available in this retrospective cohort and therefore could not be incorporated into the risk prediction models. Finally, we lacked data on cardiovascular-related readmissions, as a majority of patients were admitted to our department specifically for liver-related events. Future prospective, multicenter, large-scale cohort studies are needed to address these limitations and further explore the relationship between MetS and alcohol-associated cirrhosis.

## Conclusion

In summary, our findings indicate that MetS is prevalent among patients with alcohol-associated cirrhosis, and that these patients exhibit distinct differences in clinical indicators. Moreover, the presence of MetS is associated with an increased risk of readmission, suggesting that special attention should be paid to patients’ metabolic status and the development of complications during clinical management.

## Data Availability

The raw data supporting the conclusions of this article will be made available by the authors without undue reservation.
